# Long-term *in toto *cell tracking using lightsheet microscopy of the zebrafish tailbud

**DOI:** 10.12688/wellcomeopenres.14907.1

**Published:** 2018-12-23

**Authors:** Timothy Fulton, Martin O. Lenz, Leila Muresan, Courtney Lancaster, Elizabeth Horton, Benjamin Steventon

**Affiliations:** 1 Department of Genetics, University of Cambridge, Cambridge, CB2 3EH, UK; 2 Cambridge Advanced Imaging Center, University of Cambridge, Cambridge, CB2 3EH, UK

**Keywords:** Zebrafish, Tailbud, Axial elongation, Lightsheet, Tracking, Online Registration

## Abstract

*In toto* light-sheet imaging allows the tracking of entire growing tissues with high spatial and temporal resolution for many hours. However, this technology requires a sample to be immobilised to ensure that the tissue of interest remains within the field of view throughout the image acquisition period. We have developed a method of mounting and image capture for long-term light-sheet imaging of a growing zebrafish tailbud from the 18 somite stage through to the end of somitogenesis. By tracking the global movement of the tailbud during image acquisition and feeding this back to the microscope stage, we are able to ensure that the growing tissue remains within the field of view throughout image acquisition. Here, we present three representative datasets of embryos in which all nuclei are labelled and tracked until the completion of somitogenesis.

## Introduction

Early embryonic development is characterised by large-scale cell movements that together generate tissues of the correct shape and size. Furthermore, these highly dynamic processes must be coordinated between neighbouring tissues in order to establish the connections required to build functioning organs. Recent advances in light-sheet imaging have vastly increased the speed at which whole embryos can be imaged during development, allowing for all cells to be tracked for long time periods, a processed termed “ *in toto* imaging” ( Megason, 2009). *In toto* light-sheet imaging has allowed for single-cell tracking across multiple tissues with high time resolutions during axis formation in both mouse and zebrafish embryos ( Keller *et al.*, 2008; McDole *et al.*, 2018). Recently, this has allowed the tracking of cells in the gastrulating zebrafish from 30% epiboly to 12 somites ( Attardi *et al.*, 2018a; Shah *et al.*, 2017). In this period of development, the embryo remains stationary with most growth being derived from cellular rearrangements. In later developmental stages, however, *in toto* light-sheet imaging has previously been impossible due to the large amounts of growth and global movements of the tailbud which results in the object leaving the field of view rapidly ( Hirsinger & Steventon, 2017; Steventon *et al.*, 2016).

To follow the cell movements contributing to multi-tissue morphogenesis during posterior body elongation, we have therefore developed a mounting technique for an upright, single view scanning light-sheet microscope and online tracking tool to follow the growing tailbud of zebrafish from 18 somites through to the end of somitogenesis. The online tracking tool uses image-based registration to calculate the XYZ shift of the tailbud between the N ^th^ and N+4 ^th^ frame and then centres this object back into the field of view. With imaging every 2 minutes, registration therefore occurs every 10 minutes, which is demonstrably sufficient to permit tracking of the tailbud over an extended period of time to allow tracking of cells with single cell accuracy.

## Methods

### Mounting and imaging

**Figure 1.**
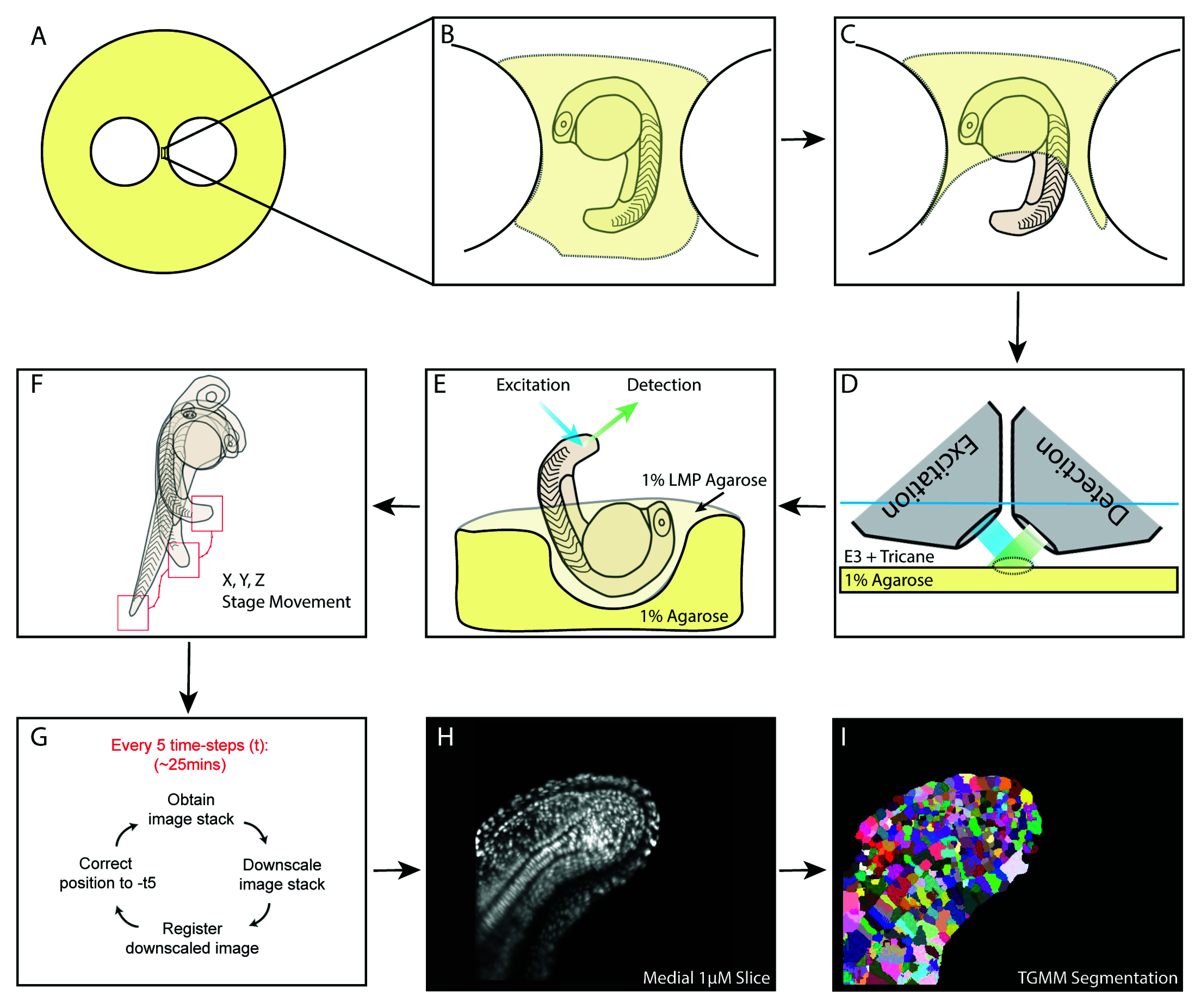
A schematic of mounting and downstream processing for 4D lightsheet imaging of the zebrafish tailbud. ( **A**) Set up of the 1% agarose base plate with two circular holes punched inside using glass rings generating a bridge onto which the embryo is mounted. ( **B**) The embryo is mounted inside a hole cut from the agarose and lined with 1% low-melting-point agarose. The embryo is fully covered with low-melting-point agarose, which is allowed to set. ( **C**) The growing tail and tailbud are then cut free from the low melting point agarose and ( **D**) placed under an upright scanning lightsheet microscope under E3 plus tricane. ( **E**) A lateral view of the mounting demonstrating the hole cut into the agarose baseplate and orientation of the embryo to permit optimal illumination and imaging by the objectives which are mounted at 45 degrees to the baseplate. ( **F**, **G**) The online mid-imaging registration software allows tracking of the centre of mass of the tailbud in both X, Y and Z dimensions. ( **H**) A medial 1-µM slice of the downsampled and registered data from the first timepoint of the acquisition which is ( **I**) segmented using the TGMM software to permit tracking of single cells.

Embryos were obtained from an incross of a heterozygous constitutive Histone 2B-fused GFP line (H2B::GFP) and screened for strong green fluorescence at 50% epiboly. Embryos were then grown to the 16 somite stage prior to mounting which is described here, and graphically, in Figure 1.

To mount the embryos, 10-cm petri dishes were filled with a 5-mm layer of 1% agarose made in E3 media. Next, two glass rings were placed in the centre of these dishes, 5 mm apart, and the agarose was allowed to set. These glass rings were then removed alongside the contained agarose, leaving a bottom layer of agarose with two holes inside, separated from one another by an agarose bridge.

On this bridge, a small embryo-sized hole was cut using size 5 forceps to allow for correct orientation of the embryo relative to the light-sheet excitation objective and detection objectives. The hole was then lined, by filling and removing 1% low-melting-point agarose made in E3.

Finally, the embryo was mounted into this hole by aspirating the dechorionated embryo in low melting point agarose and placing it into the lined hole on the petri dish. The embryo was orientated to that the embryo is ventral-side-up and laying at a 45 degree angle to the agarose layer. The agarose was then allowed to set fully before filling the dish with E3 media plus tricane methanesulfonate. Using a fine glass capillary needle, the agarose was cut away from the tail whilst leaving the anterior of the embryo fixed in place. The embryo was then imaged on the light-sheet microscope as described by Attardi *et al*. (2018a).

This mounting technique permits the embryo to be fixed in position, from the anterior agarose, which prevents the sample moving as the objectives scan the sample whilst also allowing unrestrained growth of the tissue posteriorly. This technique also ensures that the embryo tail is correctly aligned for optimal illumination from the illumination objective and viewing from the collection objective which both sit at 45 degrees to the stage.

### Post-image-acquisition processing

Following image acquisition, the data was downscaled, so that a voxel represents 1 µm ^3^, and registered to remove the visible stage movements caused by the tracking program. Finally the data was tracked using the Tracking with Gaussian Mixture Models (TGMM) software vOct-17 ( Amat *et al.*, 2014) as described in Attardi *et al.* (2018a) followed by manual validation of tracks using Mamut v0.27.0 for Fiji v1.52d ( Wolff *et al.*, 2018). Figure 1 shows a representative binary output from automatic segmentation of the three-dimensional image from the starting timeframe from which lineage inferences were made.

The complete dataset generated is available on the Image Data Resource (Attardi, 2018b)

### Ethics policies

This research has been regulated under the Animals (Scientific Procedures) Act 1986 Amendment Regulations 2012 following ethical review by the University of Cambridge Animal Welfare and Ethical Review Body (AWERB). 

## Dataset validation

Of the automatic tracks generated, a subset was validated. A detailed description of this validation is available from Attardi *et al*. (2018a). Approximately 75% of automatic tracks correctly followed a single cell to the termination of the track, with the remaining 25% requiring some level of human intervention to either correct or discard the track.

## Data availability

The imaging data as registered, downscaled .tiff, downscaled .klb files and associated tracking data, as .xml, are available from the Image Data Resource. DOI: https://doi.org/10.17867/10000117 ( Attardi *et al.*, 2018b)

Data are available under the terms of the Creative Commons Attribution 4.0 International license (CC-BY 4.0).
