# Supplementary material for: Long-term in toto cell tracking using lightsheet microscopy of the zebrafish tailbud
Source: Wellcome Open Res. 2026 Apr 16;3:163. Originally published 2018 Dec 23. [Version 3] doi: 10.12688/wellcomeopenres.14907.3 (PMC13096779; doi:10.12688/wellcomeopenres.14907.3)
Supplement: Peer review history

# Reviewer report: Reviewer response for version 3

**DOI:** 10.21956/wellcomeopenres.28822.r153453

**Published:** 2026-04-24

**Authors:** Johannes Stegmaier

**Affiliations:** 1 Machine Learning for Medical Data, Heinrich Heine University Düsseldorf Faculty of Mathematics and Natural Sciences, Düsseldorf, North Rhine-Westphalia, Germany

Thanks for the clarifications, I have no further comments.

Are sufficient details of methods and materials provided to allow replication by others?

Partly

Is the rationale for creating the dataset(s) clearly described?

Yes

Are the datasets clearly presented in a useable and accessible format?

Partly

Are the protocols appropriate and is the work technically sound?

Partly

Reviewer Expertise:

3D Image Analysis

I confirm that I have read this submission and believe that I have an appropriate level of expertise to confirm that it is of an acceptable scientific standard.

# Reviewer report: Reviewer response for version 3

**DOI:** 10.21956/wellcomeopenres.28822.r153600

**Published:** 2026-04-20

**Authors:** Zeguan Wang

**Affiliations:** 1 Massachusetts Institute of Technology, Cambridge, Massachusetts, USA

Fulton et al. describe a zebrafish embryo mounting technique and 3D position correction software that allow upright light-sheet imaging of tailbud development from the 18-somite stage through to the end of somitogenesis, a process that lasts several hours. The description and the schematic illustrations of the mounting procedure are clear and instructive. The authors also describe the image processing pipeline, provide the software information, and more importantly, openly share example raw datasets. The availability of such datasets is highly valuable, as it allows researchers to understand what such data look like in practice and to benchmark image quality. Therefore, this article can potentially serve as a useful guide and reference for researchers interested in studying zebrafish development using light-sheet imaging.

 The authors claimed that their approach achieved single-cell accuracy tracking, and that ~75% of automatic tracks correctly follow a single cell throughout the track. This claim would benefit from further clarification and supporting results and analysis. The segmentation results shown in Figure 1H–I would be strengthened by a clearer explanation of how single-cell accuracy was validated, and over which regions this validation was performed. Additionally, while the associated tracking data (xml files) are said to be available in the online repository, it is not straightforward for readers to visualize and interpret these data without further guidance. Providing instructions or example workflows for accessing and visualizing these tracking results would be a valuable addition.

 Minor comments:

 1. Fish embryos were imaged in tricaine E3 medium, presumably to reduce motion. The concentration of tricaine should be specified, and it would be helpful to comment on whether and how tricaine may affect embryonic development, particularly during long-term imaging of tailbud morphogenesis.

 2. In Figure 1H, the term “1-μM slice” is unclear and appears to be a typo (μm vs μM). This should be corrected.

 3. It would be useful to include a brief description of the light-sheet microscope configuration, including objective lenses, cameras, and key imaging parameters such as excitation intensity and exposure time. This would help readers better interpret the shared datasets and compare them with their own results.

Are sufficient details of methods and materials provided to allow replication by others?

Partly

Is the rationale for creating the dataset(s) clearly described?

Yes

Are the datasets clearly presented in a useable and accessible format?

Yes

Are the protocols appropriate and is the work technically sound?

Yes

Reviewer Expertise:

Optical Microscopy

I confirm that I have read this submission and believe that I have an appropriate level of expertise to confirm that it is of an acceptable scientific standard.

# Reviewer report: Reviewer response for version 2

**DOI:** 10.21956/wellcomeopenres.16774.r36157

**Published:** 2019-08-08

**Authors:** Johannes Stegmaier

**Affiliations:** 1 Machine Learning for Medical Data, Heinrich Heine University Düsseldorf Faculty of Mathematics and Natural Sciences, Düsseldorf, North Rhine-Westphalia, Germany

Fulton *et al.* present a method for embedding zebrafish embryos that were raised to the 16-somite stage such that tailbud development can be imaged with an upright light-sheet microscope. The pipeline involves automatic estimation of global shifts of the specimen in 3D using image registration techniques that are applied in fixed time intervals and are used to readjust the field of view of the microscope. The final registered data set is used for single cell tracking in 3D with an established software tool (TGMM by Amat *et al.*, 2014). The data note is generally well understandable and I agree with Reviewer 1 (Fiolka, 2019 ^1^) that it’s indeed nice to see that even such large-scale data sets are becoming publicly available. However, the note lacks a bit of technical detail for the involved software aspects that would make it difficult to reproduce the presented results. Moreover, I have some doubts that the segmentation/tracking quality is indeed as good as the authors describe it.

 Some more specific remarks and suggestions:

- The authors claim that an “image-based registration is used to calculate XYZ shift”. Could you comment a bit more on what registration technique was used? How is it incorporated to the imaging workflow? How much time does the registration need to align two sequential 3D images? Are there any special hardware requirements to do it in the constrained time interval?
- The segmentation result presented in Figure 1I does not look very convincing and it seems that there are many undersegmentation errors. There are a few reasons why this could happen: (1) Ideally, it’s just a visualization issue due to a limited number of label colors, (2) it could be caused by insufficient spatial resolution considering the relatively high density of nuclei at this later developmental stage or (3) the parameterization of the TGMM algorithm was not performed properly. Of course, it could also be a combination of all three issues. Can you comment on this and explain how/if the parameters of the method were adjusted for that particular data set?
- Mini note: The type of image visualized in Figure 1I is usually termed “label image” or “label map” rather than “binary output” (which would only distinguish background vs. foreground).
- To provide an easier assessment of the segmentation quality presented in Figure 1I, you could superimpose label boundaries on top of the raw image shown in Figure 1H. This would also allow to see if the apparent undersegmentations are actually only a visualization issue or indeed an error of the segmentation. You could simply compute the intensity gradient of the label image, binarize it by setting everything above zero to 1 and then superimpose the raw image with the cell region boundaries.
- For the tracking validation you mention “75% of automatic tracks correctly followed a single cell to the termination of the track”. Does this mean a track spans the entire duration of the experiment or would a track that only connects two time points successfully also be considered as a correct automatic track? In Attardi *et al.* (2018a), the tracking validation seems to be described only for the 30%-75% epiboly stage – how was selection of the subset of cells used for validation of the tailbud segmentation/tracking performed?
- Availability of the registration software is unclear: is this a module that can be easily added to any upright microscope setup/software? If yes, where can it be obtained from?
- Availability of the data seems to be provided. However, I only managed to view the data online but was not allowed to download anything else than PNG images of the current view. The tracking files are listed in the tables overview, but it was not obvious how those files can be downloaded from the repository. Maybe comment on how to actually obtain the data in case there are any special things to consider.

Are sufficient details of methods and materials provided to allow replication by others?

Partly

Is the rationale for creating the dataset(s) clearly described?

Yes

Are the datasets clearly presented in a useable and accessible format?

Partly

Are the protocols appropriate and is the work technically sound?

Partly

Reviewer Expertise:

3D Image Analysis

I confirm that I have read this submission and believe that I have an appropriate level of expertise to confirm that it is of an acceptable scientific standard, however I have significant reservations, as outlined above.

## References

[1] : Referee Report For: Long-term in toto cell tracking using lightsheet microscopy of the zebrafish tailbud [version 1]. *Wellcome Open Research* .2019;3(163) : Reference source 10.12688/wellcomeopenres.14907.3PMC1309677942022023

# Response

**Published:** 2020-05-12

**Authors:** Benjamin Steventon

**Affiliations:** University of Cambridge, UK

We thank both reviewers again for their time in reviewing our Data Note and are pleased they agree with us the importance of publishing short descriptive articles of large datasets and the importance of making them freely available to the community.

The reviewer is indeed correct in saying that parts of the image are under segmented. These regions tend to be the anterior regions of the tail and therefore outside of the region of interest for our published work in Attardi & Fulton et al. (2018a). The parameter search used to set up the initial image segmentation was not exhaustive however, parameters were identified that gave sufficiently high image segmentation to the dorsal posterior wall of the tailbud, where we wanted to focus our studies. The automatic segmentation was not the end of the pipeline in our image analysis as we then subset tracks from this dorsal posterior wall region and manually validated whether the track followed a single cell until the termination of the track. As no conclusions in Attardi & Fulton et al (2018a) were drawn from automatic tracks which had not been manually validated, we felt it was not required to undertake an exhaustive parameter search. The reviewer is correct in stating that the track may not terminate at the end of the experiment, and an accurate track would be any one which did not switch cell along the track duration. We have better described the methods in the main text. 

We thank the reviewer for seeking further clarity regarding the registration process. The microscope is controlled using an in-house developed Labview software. Initial image handling within the acquisition software was integrated into the bespoke software utilising Matlab scripts. The images stacks were downsampled immediately after acquisition to a voxel size of 1um^3. The downsampled stacks were then used to estimate the relative translative offset between the Nth and (N+4)th stack using image phase correlation for 3D registration. The computation time for determining the shift between two image stacks is about 4-5 sec on the microscope workstation. In combination with the fact that the calculation and successive position correction is performed only every 10 min, this does not slow down the acquisition. The above mentioned phase correlation based Matlab scripts were also used for the image registration post acquisition and is applied to every timeframe. These scripts are available from the authors upon request. 

The full dataset is available from the Image Data Repository. Detailed instructions on how to download the raw files from the IDR are available on their website, linked here https://idr.openmicroscopy.org/about/download.html. We also thank the reviewer for noting the mislabelling of panel 1I, we have corrected this in the text.****

# Reviewer report: Reviewer response for version 1

**DOI:** 10.21956/wellcomeopenres.16250.r34929

**Published:** 2019-04-29

**Authors:** Reto Fiolka

**Affiliations:** 1 Department of Cell Biology, University of Texas Southwestern Medical Center, Dallas, TX, USA

In this note, Fulton *et al.* detail their workflow to image and track nuclei in the tailbud of a growing Zebrafish. The methods detail a novel way of mounting the Zebrafish in 1% Agarose such that the tail is not embedded and can be imaged in an inverted light-sheet geometry. To me this is the biggest innovation in this note; the other steps are using tools described previously by the authors and other groups.

 I appreciated that there is an online data repository where raw data can be viewed. I think this is very valuable for the community, as too often raw data is not available and only finished and processed data is shown. As such, I think this can be very useful to other groups to compare their image quality.

 Some questions and comments:

- The authors claim that tracking of the movement of the sample and growth have not previously been accounted in acquisition software. However, such capabilities are described in the Autopilot method by the Keller lab. A description of the algorithm for centering the embryos and adjusting the imaging volume (in conjunction with adjusting the optical model of the AutoPilot itself) is available on pages 24-26 in McDole *et al.* (2018 ^1^). This publication should be cited and it would be great if the authors could comment if they think Autopilot could be used here too.
- Upon looking at the online data, it looks that in some cases, not the whole image is sharp – some nuclei are blurry. Is this a problem for the tracking algorithm and would a multiview implementation (i.e. diSPIM) help?
- For Figure 1 A-D, coordinate axes would be helpful. I was not fully sure if E shows the same plane as A-C.

Are sufficient details of methods and materials provided to allow replication by others?

Partly

Is the rationale for creating the dataset(s) clearly described?

Yes

Are the datasets clearly presented in a useable and accessible format?

Yes

Are the protocols appropriate and is the work technically sound?

Yes

Reviewer Expertise:

Optical microscopy

I confirm that I have read this submission and believe that I have an appropriate level of expertise to confirm that it is of an acceptable scientific standard.

## References

[1] : In Toto Imaging and Reconstruction of Post-Implantation Mouse Development at the Single-Cell Level. *Cell* .2018;175(3) : 10.1016/j.cell.2018.09.031 859-876.e33 10.1016/j.cell.2018.09.031 30318151
